# Assessment of HIV/AIDS comprehensive correct knowledge among Sudanese university: a cross-sectional analytic study 2014

**DOI:** 10.11604/pamj.2016.24.48.8684

**Published:** 2016-05-11

**Authors:** Abdulateef Elbadawi, Hyder Mirghani

**Affiliations:** 1 Community Medicine Department, Faculty of Medicine, University of Tabuk, Saudi Arabia; 2 Medical Department, Faculty of Medicine, University of Tabuk, Saudi Arabia

**Keywords:** HIV, AIDS, comprehensive, knowledge, misconception, university students

## Abstract

**Introduction:**

Comprehensive correct HIV/AIDS knowledge (CCAK) is defined as correctly identify the two major ways of preventing the sexual transmission of HIV, and reject the most common misconceptions about HIV transmission. There are limited studies on this topic in Sudan. In this study we investigated the Comprehensive correct HIV/AIDS knowledge among Universities students.

**Methods:**

A cross-sectional analytic study was conducted among 556 students from two universities in 2014. Data were collected by using the self-administered pre-tested structured questionnaire. Chi-square was used for testing the significance and P. Value of ≥ 0.05 is considered as statistically significant.

**Results:**

The majority (97.1%) of study subjects have heard about a disease called HIV/AIDS, while only 28.6% of them knew anyone who is infected with AIDS in the local community. Minority (13.8%) of students had CCAK however, males showed a better level of CCAK than females (OR = 2.77) with high significant statistical differences (P. Value = 0.001).

**Conclusion:**

Poor rate of CCAK among university students is noticed, especially among females. Almost half of students did not know preventive measures of HIV, nearly two thirds had misconception, about one third did not know the mode of transmission of HIV.

## Introduction

The understanding of the epidemic context in Sudan has increased substantially. Evidence from the 2011 integrated bio-behavioral Survey (IBBS) among key populations, 2010 ANC surveillance rounds and routine programmatic data from VCT and PMTCT sites shed more light on the type of HIV epidemic that exists in Sudan [1]. The data suggests a concentrated HIV/STI epidemic among key populations in specific geographical foci in Sudan. The HIV epidemic in the general population is still low [2]. All the rounds of ANC surveillance, population-based surveys, and HIV testing data indicate an HIV prevalence of less than 1%. In 2013, HIV prevalence among adult population (15 – 49) is estimated at 0.31% – 0.42% translating to 67,830 (59,731 – 80,698) people living with HIV [3]. The annual estimations of new HIV infections and AIDS deaths are 7,032 (5,631 – 9922) and 4,797 (4,163 – 5,623) respectively [4]. In 2013 the Percentage of young women and men aged 15–24 who both correctly identify ways of preventing the sexual transmission of HIV and who reject major misconceptions about HIV transmission was estimated at 6.7% (11.1% in male and 5.3% in female) [4]. The status of the epidemic is not expected to remain static with the challenging socio-political and economic changes that occurred in the post-secession era. Also, the existent low HIV knowledge and behavioral practices among key and general population if not addressed could potentially increase population's HIV vulnerability and increase transmission. No new surveys/studies were carried out during this reporting period to compare any change or trends in knowledge and behavior for general population since the last 2010 Sudan Household [4]. It is important to note that HIV comprehensive knowledge of the general population is much lower 6.7% [5]. HIV is the leading cause of burden in men aged 30-44 and women in the 25-44 age group [6]. Sudan is a big country taking around 2% of the earth surface, with open borders with many of the African HIV belt countries. Justifications of conduction of the current study include the following facts. Firstly the existent of low HIV knowledge and behavioral practices among key and general population if not addressed could potentially increase population's HIV vulnerability and increase transmission. Secondly the knowledge is the main component of the behavioral change in both general and key population, and it is important to note that HIV comprehensive knowledge of the general population in Sudan was estimated at only 6.7% [3]. Last but not least no new surveys/studies were carried out during this reporting period to compare any change or trends in HIV knowledge and behavior for general population since the last 2010 Sudan Household Survey, thus we conducted this research in which we thought to assess the change of HIV comprehensive correct knowledge among Sudanese University students in the year 2014, in which low rate of Comprehensive correct HIV/AIDS knowledge was evident specially among women, almost half of students did not know preventive measures of HIV, nearly two thirds had misconception, about one third did not know the mode of transmission of HIV (Table 1).

**Table 1:** Shows the HIV knowledge among study subjects

Parameter	All subjects (n = 556) N (%)	Female (n = 256) N (%)	Male (n = 300) N (%)	P. Value	M: F OR
**General HIV information:**					
Subjects who ever heard about a disease called AIDS	540 (97.1)	247(96.5)	293(97.7)	0.406	1.525
Subjects who know anyone who is infected with AIDS	159 (28.6)	71 (27.7)	88 (29.3)	0.678	1.082
**Knowledge of HIV transmission:**					
**Subjects who identify that HIV infection can be transmitted:**					
Through vaginal intercourse	396 (71.2)	200(78.1)	196(65.3)	0.001	0.528
Through anal intercourse	465 (83.6)	193(75.4)	272(90.7)	0.001	3.171
Through transfusion with contaminated blood	394 (70.9)	209(81.6)	185(61.7)	0.001	0.362
Through shared injection needle	373 (67.1)	211(82.4)	162(54.0)	0.001	0.250
**Misconception regarding HIV transmission:**					
**Subjects who identify that HIV infection cannot be transmitted**					
Through Mosquito bites	299 (53.8)	112(43.8)	187(62.3)	0.001	0.470
By eating with an HIV-infected person	326(58.6)	71 (27.7)	255(85.0)	0.001	0.068
**Another misconception:**					
Subjects who Identify, that healthy looking person could be infected with HIV	180 (32.4)	138(53.9)	42(14.0)	0.001	0.139
**Knowledge regarding HIV prevention methods:**					
**Subjects who identify that HIV infection can be prevented by:**					
Consistent condom use	226 (40.6)	102(39.8)	124 (41.3)	0.722	1.064
Having only one faithful uninfected partner	327 (58.8)	76 (29.7)	251 (83.7)	0.001	12.132
**Comprehensive Correct HIV/AIDS knowledge**					
Subjects who Had comprehensive correct HIV/AIDS Knowledge	77 (13.8)	20 (7.8)	57 (19.0)	0.001	2.77

## Methods

A cross-sectional analytic study was conducted in which 556 university students were enrolled. Three stages were used for selection the study subjects. The first stage was selection of two universities out of nine at Khartoum-Sudan. The second stage includes choosing two faculties out of thirteen per each university. The last stage was selecting the third and fourth-year students in each faculty. Simple random sampling technique was adopted in all stages. All the students (males and females) who attending the classes during the study period were invited to join the study, 556 out of 750 students responded to the questionnaire giving a response rate of 74.13%. The male: female ratio was 1.2:1. Data was collected by using a self-administered pre-tested structured questionnaire that contains general information and HIV knowledge questions. Facilitators from the four randomly chosen faculties attended while the participants were responding to the questionnaire for any explanations, and the principal investigator arranged an orientation meeting with the students and briefed them about the research objectives and how to fill the questionnaire.

### Measures

Comprehensive correct knowledge of HIV/AIDS (CCAK) had the following components; identifying that using condoms consistently and limiting sex to one faithful, uninfected partner are two ways to prevent HIV/AIDS transmission; rejecting common misconceptions that mosquitoes transmit HIV/AIDS and sharing food with an infected person transmits HIV/AIDS, and knowing that a healthy-looking person can have HIV/AIDS.

### Statistics

The Statistical package for the Social Sciences software SPSS version 20 was used for data analysis. The Chi-square test was used for comparison between male and female students regarding their HIV knowledge components. P. Value equal or less than 0.05 was accepted for statistical significance.

### Ethical considerations

Ethical issues were carefully followed conforming the medical research ethics. Approvals were obtained from Universities administration. Privacy, confidentiality, and anonymity were ensured. Written informed consent was used for taking informant's willingness to participation.

## Results

The majority (97.1%) of study subjects have heard about a disease called HIV/AIDS, while only 28.6% of them knew anyone who is infected with AIDS in the local community. Only (13.8%) of students had CCAK and significant differences (P. Value = 0.001) was found between Male and female in their level of CCAK. Males showed a better level of CCAK than females (OR = 2.77). Regarding the modes of HIV transmission, vaginal intercourse was identified by 78.1% of female students and 65.3% of male students (P. Value = 0.001). Anal intercourse was identified by 75.4% of female students and 90.7% of male students (P. Value = 0.001). Transfusion of contaminated blood was identified by 81.6% of female students and 61.7% of male students (P. Value = 0.001). Shared injection needle was identified by 82.4% of female students and 54.0% of male students (P. Value = 0.001). HIV transmission misconceptions were also examined during the study. Only 43.8% of female students and 62.3% of male students identify that HIV cannot transmit by mosquito bites (P. Value = 0.001). Only 27.7% of female students and 85.0% of male students identify that eating with the HIV-infected person does not transmit HIV (P. Value = 0.001). Only 14% of male students and 53.9% of female student identify that healthy looking person may have HIV (P. Value = 0.0001). The researcher also examined the level of knowledge about HIV preventing methods. Consistent condom use was identified by only 39.8% of female students and 41.3% of male students (P. Value = 0.722). Having one faithful uninfected partner was identified by only 29.7% of female students and 83.7% of male students (P. Value = 0.001) and (OR = 12.13).

## Discussion

The data in the present study indicated a poor comprehensive correct knowledge among Sudanese University students. Generally females had more knowledge about the transmission of HIV, while males knew more about misconception. The poor rate of CCAK among university students is noticed, and it is not expected because of their higher education. The explanation may be that CCAK was calculated by five components and failure to answer correctly all the questions resulted in negative CCAK result. A similar study conducted in Ethiopia in 2011, showed a low rate of HIV knowledge among in-school adolescents [7]. In 2012, another study conducted in Tanzania showed a high level of knowledge among university students in spite of spreading of misconceptions towards HIV transmission [8]. In the current study 97.1% of students ever heard of AIDS, similarly researchers from Ghana concluded that 97.9% of college students had heard about AIDS [9] General information of HIV is available for all communities by many means such like media and health programs [10, 11]. All modes of transmission were identified by considerable percentage among both male and female students. Previous literature reported gender differences in the mode of transmission of HIV infection with more knowledge among females [12, 13], in the current study female students had higher rates than male students in all modes of HIV transmission except the anal intercourse; this point needs more focus in health education campaigns and programs putting in mind it is culture sensitive issue in Sudanese female community. Misconception about HIV may create stigma to people suffering from this serious disease that leads to double jeopardy on their lives. Misconception may be a major barrier to control and prevention of AIDS [14–16]. One of the most important component in HIV comprehensive knowledge is the transmission misconceptions, both mosquito bites and eating with HIV-infected person misconceptions were poorly identified by all student, but male students had a higher knowledge than female ones, in particular, component. The poor performance in HIV transmission misconceptions had the main role in lowering CCAK level rate in all students. A study published in China [17] showed that 26.5% of participants believed that HIV can be transmitted by sharing meals and lower than the present data in which 41.4% of University students did not know that HIV is not transmitted by eating with those with AIDS. Male students also had higher rates than female in HIV prevention methods that contain both consistent condom use and having one faithful uninfected partner. Sudan society is highly conservative and had specific sensitivity about sexual issues and practicing sex with men due to religious and tradition issues, also it is rare for parents to discuss sensitive topics with family members, the unwanted loss of this effective protective shield can be compensated for by social media which was proven to be a very effective way of compacting AIDS [18]. Female students should be targeted by awareness raising activity focused on HIV transmission misconceptions and HIV prevention methods. The current data strength is that it was conducted among special sector that can have positive influence on the community if they are provided with the right information, the participation of facilitators in data collection could minimize but not totally illuminate the bias. Unlike previous researchers we compared both sexes for knowledge putting into consideration the social and religious background of our community, that if ignored may create difficulty in delivering the right knowledge and hence prevention of HIV. Although the current study reflects the situation of comprehensive HIV knowledge among Sudanese university students, the result cannot be generalized because it was conducted at two Universities. The reliance on self-reported questionnaire is more prone to subjectivity. A larger scale study is needed to support this information.

## Conclusion

The data in the present study indicated a poor comprehensive correct knowledge among Sudanese University students. Generally, females had more knowledge about the transmission of HIV, and less awareness about misconception.

**Recommendations:** Raising the awareness about this spreading morbid disease by Including HIV education among school curriculum is highly needed to prevent transmission and reduce the great pressure we put on those suffering the disease by misconception.

### What is known about this topic

- HIV knowledge among most at risk populations was addressed by few studies in Sudan;
- Sudan had low to concentrated level of HIV epidemic;
- HIV knowledge is the first step to change the risky behaviors among both general and high risk populations.

### What this study adds

- Current study provides important information about level of comprehensive HIV knowledge among University students specially the females who are more prone to the disease;
- The results of this study will guide the HIV interventions among general population and help the Sudan AIDS program in future planning;
- Most of the previous studies focused on high-risk population, the current study will be the first study addressing level of the HIV knowledge among general population in Sudan.
